# Investigation of the Effects of Fosfomycin in Kidney Damage Caused by CLP-Induced Sepsis

**DOI:** 10.3390/life15010002

**Published:** 2024-12-24

**Authors:** Ilknur Esen Yildiz, Tolga Mercantepe, Ilkay Bahceci, Medeni Arpa, Sule Batcik, Yasin Yildiz, Levent Tumkaya

**Affiliations:** 1 Department of Infectious Diseases and Clinical Microbiology, Faculty of Medicine, Recep Tayyip Erdogan University, 53100 Rize, Turkey; 2 Department of Histology and Embryology, Faculty of Medicine, Recep Tayyip Erdogan University, 53100 Rize, Turkey; tolga.mercantepe@erdogan.edu.tr; 3 Department of Medical Microbiology, Faculty of Medicine, Recep Tayyip Erdogan University, 53100 Rize, Turkey; ilkay.bahceci@erdogan.edu.tr; 4 Department of Medical Biochemistry, Faculty of Medicine, Recep Tayyip Erdogan University, 53100 Rize, Turkey; medeni.arpa@erdogan.edu.tr; 5 Department of Anaesthesiology and Reanimation, Faculty of Medicine, Recep Tayyip Erdogan University, 53100 Rize, Turkey; sule.batcik@erdogan.edu.tr; 6 Department of Pediatrics, Faculty of Medicine, Recep Tayyip Erdogan University, 53100 Rize, Turkey; yasin.yildiz@erdogan.edu.tr; 7 Department of Histology and Embryology, Faculty of Medicine, Ondokuz Mayıs University, 55139 Samsun, Turkey

**Keywords:** fosfomycin, inflammation, CLP, kidney, rat, sepsis

## Abstract

Sepsis, a life-threatening condition characterized by dysregulated host responses to infection, often leads to multi-organ dysfunction, including kidney injury. Kidney damage in sepsis can have severe consequences and is associated with high mortality rates. This study aimed to investigate the potential therapeutic effects of fosfomycin (FOS), a broad-spectrum antibiotic with immunomodulatory properties, on kidney damage induced by cecal ligation and puncture (CLP)-induced sepsis in a rodent model. In total, 24 rats were randomly divided into three groups. Group 1 (*n* = 8), the healthy control group (C), received a single dose of 0.9% NaCl (saline) solution via an intraperitoneal (i.p.) route. To group 2 (*n* = 8), the CLP group, CLP-induced sepsis was applied without medication, and a single dose of 0.9% NaCl (saline) solution was applied i.p. before induction. To group 3 (*n* = 8), the CLP + FOS (500 mg/kg) group, a single dose of 500 mg/kg FOS was administered i.p. before sepsis induction. The effects of fosfomycin on kidney function, histopathological changes, inflammatory markers, oxidative stress, and apoptosis were assessed. In the fosfomycin-treated group, the histological analysis results demonstrated reduction in kidney tissue damage and inflammation. Additionally, fosfomycin attenuated the upregulation of pro-inflammatory cytokines and reduced oxidative stress markers in kidney tissue. Furthermore, fosfomycin treatment was associated with a decrease in apoptotic cell death in the kidney. These findings suggest that fosfomycin may have a protective effect on kidney damage caused by CLP-induced sepsis. The potential mechanisms underlying this protection include the modulation of inflammation, reduction of oxidative stress, and inhibition of apoptosis.

## 1. Introduction

Sepsis is defined as life-threatening organ dysfunction, and kidneys are one of the important organs affected in this circumstance. Acute renal failure (acute kidney injury, AKI) caused by sepsis itself or the antimicrobial used in treatment has significant effects on the course of the disease. AKI is detected in almost half of the septic patients, and the development of it has been associated with increased mortality. AKI is among the leading causes of death, especially in intensive care units. AKI resulting from sepsis is seen as one of the critical health problems worldwide [1,2,3,4,5].

In the case of kidney damage in sepsis, pro-inflammatory cytokines migrate to the kidney tissue as an immune response. It has been shown in numerous previous studies that the inflammatory process, which begins with an increase in pro-inflammatory cytokine levels, decreases with different treatments [6,7,8,9,10]. However, to our knowledge, fosfomycin (FOS) has not been studied in kidney damage caused by cecal ligation and puncture (CLP)-induced sepsis. In this study, the levels of interleukin-1ß (IL1ß) and interleukin-6 (IL-6), which are pro-inflammatory mediators that play an active role in the inflammatory process, were evaluated after FOS application.

The increase in the release of pro-inflammatory cytokines also causes an increase in oxidative stress in the kidney tissue and endothelium, resulting in an increase in the level of reactive oxygen species (ROS). Being one of the biomarkers of cell damage in experimentally induced sepsis, malondialdehyde (MDA) is one of the most important parameters showing the oxidant effect that occurs with oxidative stress. Previous studies have shown an increase in MDA levels in the kidney with an increased oxidative stress in sepsis [5,6,11,12]. Separately, under oxidative stress, antioxidant mechanisms come into play to reduce oxidative damage and protect the tissue. The most significant of these is glutathione (GSH). GSH is an antioxidant containing a thiol group (-SH) responsible for protecting tissue against the effects of ROS. In the literature, it is shown that the GSH level decreases in case of any tissue damage, such as in sepsis; in studies where tissue damage was prevented, GSH levels were shown to increase [6,7,11,12,13]. Our previous study revealed that FOS has antioxidant effects in the rat lung cortex [5]. In this study, the antioxidant effects of FOS were evaluated to prevent damage to kidney tissue caused by oxidative stress that was caused by CLP-induced sepsis. Another critical mechanism that plays a role in tissue damage in sepsis is apoptosis. It is thought that a dysregulated immune response in the course of sepsis causes apoptotic consequences, and side effects related to sepsis treatment also contribute to this negative course [14]. In the study, it was also examined whether FOS had anti-apoptotic effects by looking at the levels of 8-hydroxy 2-deoxyguanosine and cleaved caspase-3. Recent research has suggested that fosfomycin may possess additional therapeutic potential due to its ability to modulate immune responses and diminish inflammation [5]. While its antibacterial properties are well established, its impact on the pathophysiological processes that underlie sepsis-induced kidney damage remains an area of active investigation.

This study seeks to investigate the effects of fosfomycin on kidney damage induced by cecal ligation and puncture (CLP)-induced sepsis, focusing on the key mechanisms of oxidative stress, inflammation, and apoptosis. By unraveling the potential renoprotective properties of fosfomycin in the context of sepsis, we aim to contribute to understanding the pathogenesis of sepsis-induced kidney injury and explore novel therapeutic avenues for its management.

## 2. Materials and Methods

### 2.1. Experimental Animals

A total of 24 male Sprague-Dawley (SD) rats with an average weight of 350 ± 30 g were used in the study. All animals were monitored in accordance with the principles outlined in the National Research Council Guide for the Care and Use of Laboratory Animals. All animals were kept in standard plastic cages with sawdust floors at 21–23 °C and 55 ± 10% humidity under controlled lighting conditions (12 h dark: 12 h light) throughout the study. Unlimited access to standard feed and tap water was allowed. Approval for the study was also received by the local ethics committee. The entire experimental protocol was carried out at Recep Tayyip Erdoğan University Experimental Animal Application Center (Approval Number and Date: 2020/08-28.02.2020)

#### Experimental Protocol

Each group was randomly divided into three groups of 8 rats with similar average weights. Group 1 (*n* = 8), healthy control group (C), received a single dose of 0.9% NaCl (saline) solution via an intraperitoneal (i.p.) route. To group 2 (*n* = 8), cecal ligation and puncture group (CLP), CLP-induced sepsis was applied without any medication, and the rats also received a single dose of 0.9% NaCl (saline) solution i.p. before induction. To group 3 (*n* = 8), CLP + FOS (500 mg/kg) group, a single dose of 500 mg/kg FOS was administered i.p. before sepsis induction.

### 2.2. Cecal Ligation and Puncture-Induced Sepsis Model

Sepsis was induced by the CLP-induced sepsis model in rats previously described by Rittirsch D. et al. [15]. All surgical procedures were performed under sterile conditions. Rats were anesthetized with a 50 mg/kg ketamine HCL injection and 10 mg/kg xylazine HCL. After testing when the rats were under anesthesia, an incision of approximately 2.5–3 cm was made in the midline of the abdomen. The internal organs and cecum were separated through this small incision, and the cecum was tied with 3/0 silk suture from the distal part of the ileocecal valve. Similar to previous studies, two holes were made in the distal cecum, and the contents of the cecum were brought into contact with the peritoneum [5,15]. The wound was washed with 1% lidocaine for analgesia and then closed with two layers of sterile silk 4/0 suture. The experiment was terminated 16 h after the relevant processes were completed [16]. At the end of the experiment, the rats were euthanized by applying a high dose of anesthetic. One of the kidney tissues was stored at −80 °C for use in biochemical studies. The other one was placed in 10% neutral formalin.

### 2.3. Biochemical Procedure

#### Homogenization of Kidney Tissue

A total of 100 mg of rat kidney tissue was homogenized by adding 1 mL of homogenization solution (20 mM 1 L sodium phosphate + 140 mM potassium chloride with pH 7.4), and the supernatant was obtained by centrifuging at 800× *g* at 4 °C for 10 min [17].

### 2.4. Malondialdehyde (MDA) Analyses

The determination was made according to the Ohkawa et al. method. The results were calculated as nmol/g tissue [18].

### 2.5. Glutathione (GSH) Analyses

–SH groups were determined with Ellman reagent. a total of 100 µL 3 M Na_2_HPO_4_ and 25 µL DTNB were placed on 25 µL supernatant (4 mg DTNB was prepared in 10 mL 1% sodium citrate solution), and the yellow color formed after gentle shaking was read in the spectrophotometer at 412 nm. The results were determined with the prepared 1000–62.5 µM reduced glutathione standard graph and calculated as mmol/g of tissue.

### 2.6. IL-1β ve IL6 Analyses

IL-1β and IL-6 analyses in rat kidney tissue were measured using rat IL-1β and IL-6 ELISA kits (Catalog nos. E0119Ra and E0135Ra, respectively, Bioassay Technology Laboratory, Shanghai, China). Sensitivity values were 10.23 pg/mL and 0.052 ng/mL, respectively, and standard curve ranges were 0.052 ng/L and 0.1–40 ng/L, respectively. Tissue homogeneity was produced in a laboratory setting in line with the manufacturer’s recommendations. Tissue IL-1β and IL-6 concentrations in all samples were calculated in triplicate.

### 2.7. Statistical Analysis

Statistical analyses were performed with IBM SPSS Statistics v.18 (IBM Corp. IL, Chicago, IL, USA). Descriptive categorical variables were reported as *n* (%). Continuous numerical variables were expressed as median (minimum–maximum). Differences in continuous numerical variables between groups were evaluated with Kruskal–Wallis analysis. The data obtained as a result of histopathological and immunohistochemical analyses in our study were analyzed with skewness–kurtosis, Q–Q plot, Shapiro–Wilk and Levene’s tests. Non-parametric data were calculated as median and 25–75% interquartile range. Differences between groups were evaluated using Kruskal–Wallis and Tamhane T2 tests. *p* < 0.05 was considered statistically significant.

### 2.8. Histopathological Analyses

Kidney tissue samples removed from rats were fixed in a 10% neutral formalin (Sigma-Aldrich, Merck GmbH, Darmstadt, Germany) solution for 48 h. The fixed kidney tissue sections were dehydrated by passing them through an increasing series of ethanol (30%, 50%, 70%, 80%, 90%, 96%, and 100%, Merck GmbH, Darmstadt, Germany) using a tissue-tracking device (Citadel 2000, Thermo Scientific, Darmstadt, Germany), and then they were kept in two series of xylene solutions (Merck GmbH, Darmstadt, Germany) to make them clear. In the next stage, kidney tissue samples that underwent routine histological follow-up were embedded in tissue-embedding cassettes using a tissue-embedding device (Leica, EG1150, Wetzlar, Germany) using hard paraffin (Merck GmbH, Darmstadt, Germany). Kidney tissue was cut into 4–5 µm thick sections from paraffin blocks using a rotary microtome (Leica RM2525, Wetzlar, Germany) and stained with hematoxylin–eosin (Harris hematoxylin–eosin G Merck GmbH, Darmstadt, Germany) using a closed-system tissue-staining device (Leica ST5020, Wetzlar, Germany). The obtained kidney tissue preparations were analyzed using a light microscope (Olympus BX51, Olympus Corp, Tokyo, Japan) with a digital camera attachment (Olympus DP71, Olympus Corp, Tokyo, Japan), and photographs were taken.

### 2.9. Immunohistochemical (IHC) Analysis

In our study, 8-hydroxy 2-deoxyguanosine (8-OHdG, ab48508, Abcam, Cambridge, UK), a major product of DNA oxidation, and cleaved caspase-3 primary antibody (ab2302, Abcam, UK), which plays a key role in the regulation of apoptosis and is an irreversible caspase cascade, were used. Secondary antibody kits compatible with primary antibodies were used. Primary and secondary antibodies were applied in a fully automatic closed system with the Leica Bond Max IHC/ISH stainer device (Leica microsystem, Melbourne, Australia), in accordance with the manufacturer’s instructions for use.

### 2.10. Semi-Quantative Analyses

In our study, considering the findings of degenerative renal corpuscle, necrotic tubules, intraluminal necrotic cellular debris, and excessive protein exudation in H&E-stained sections in histopathological analysis, kidney histopathological damage scoring (KHDS) was scored as in Table 1 [19,20,21,22]. Immunopositive cells in the kidney sections staining with cleaved caspase-3 and 8-OHdG primary antibodies were scored as shown in Table 2 [23]. For semi-quantitative analyses, 30 different areas randomly selected from the kidney tissue sections of each rat were scored under a light microscope under an ×40 objective. Scoring was carried out by two histopathologists, who were blinded to the study groups.

## 3. Biochemical Analysis Results

When total GSH and MDA levels were examined, higher MDA levels were detected in the CLP-induced septic group compared to the control group. In the CLP + FOS group, this increase was observed to decrease to the control group levels. However, these changes were not statistically significant (Table 3, *p*: 0.176).

The GSH level was statistically significantly higher in the CLP + FOS group than in the control group and the CLP-induced septic group. (Table 1, *p*: 0.040, *p*: 0.016, respectively) There was no significant difference in terms of GSH between the control and CLP-induced septic group.

When IL6 levels were examined between the groups, it was seen that the CLP-induced septic group had higher IL-6 levels than the control group. The CLP + FOS group had lower IL6 levels than the control group. However, these changes were not at the level of statistical significance (Table 3, *p*: 0.707).

IL-1β, one of the main pro-inflammatory cytokines, was another important parameter evaluated in the present research. Lower IL-1β levels were found in the CLP + FOS group compared to the control group; however, this difference was not statistically significant (Table 3, *p*: 0.463).

## 4. Histopathological Analysis Results

**Figure 1:**
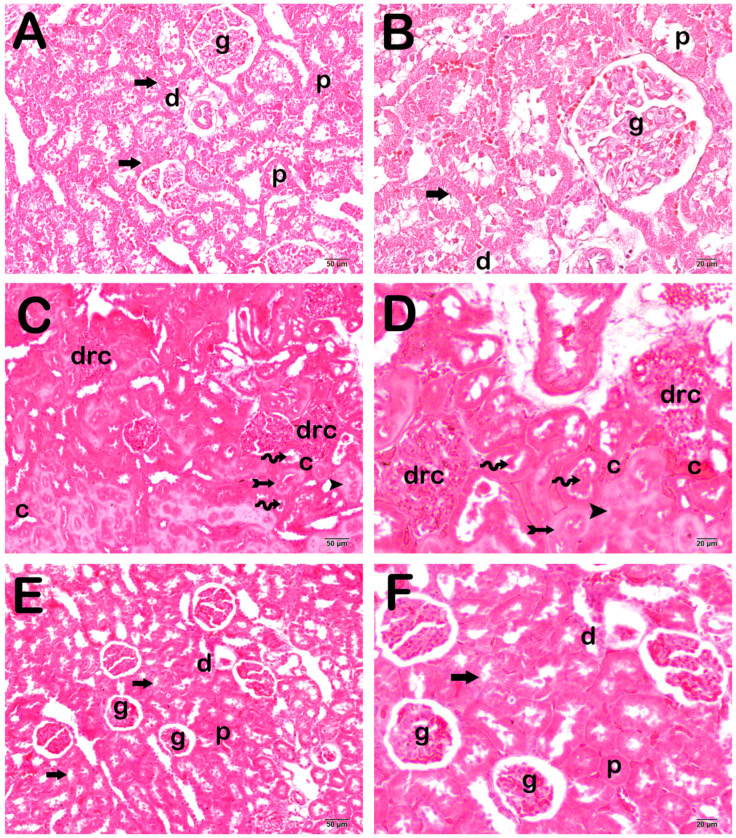
Representative light microscopic image of kidney tissue sections stained with hematoxylin and eosin. **A(x20)-B(x40):** When the sections of the control group are examined under a light microscope, renal corpuscles and renal tubules containing normal glomeruli are observed. It was noteworthy that the brush border structure in the proximal tubules (p) was typical (arrow) (KHDS: 0 (0–0)). **C(x20)-D(x40):** In the CLP application group, degenerative renal corpuscles (drc) and intraluminal necrotic cellular debris accumulation in diffuse necrotic tubules (spiral arrow) are observed (arrowhead). There was loss of brush border structures in the necrotic epithelial cells of the proximal tubule. Excessive protein exudation (arrowhead) and vascular congestions (c) are observed in intertubular areas (KHDS: 9 (9–10)). **E(x20)-F(x40):** It is observed that degenerative renal corpuscles, widespread necrotic tubules and intraluminal necrotic cellular debris accumulation decreased in the FOS treatment group. In addition, glomerular and renal tubular epithelial cells with typical structures are observed here and there (KHDS: 4.5 (4–6)). Glomerulus (g), proximal tubule (p), distal tubule (d), brush border (arrow).

When the kidney tissue sections were examined under a light microscope, we observed that the renal corpuscles and proximal and distal tubules were in normal structure in the control group (Figure 1A,B; Table 4; KHDS: 0 (0–0)). On the other hand, in the CLP-induced septic group, there were degenerative renal corpuscles and widespread necrotic tubules. In addition, there were intraluminal necrotic cellular debris structures in the renal tubules. We observed excessive protein exudation and vascular congestions in intertubular areas (Figure 1C,D; Table 4; KHDS: 9 (9–10)). On the contrary, we found that the number of degenerative renal corpuscles and necrotic tubules in the kidney parenchymal tissue decreased in the sections of the FOS treatment group. We observed that excessive protein exudation and vascular congestion were reduced in peritubular areas (Figure 1E,F; Table 4; KHDS: 4.5 (4–6)).

### Semi-Quantitative Analysis

**Figure 2:**
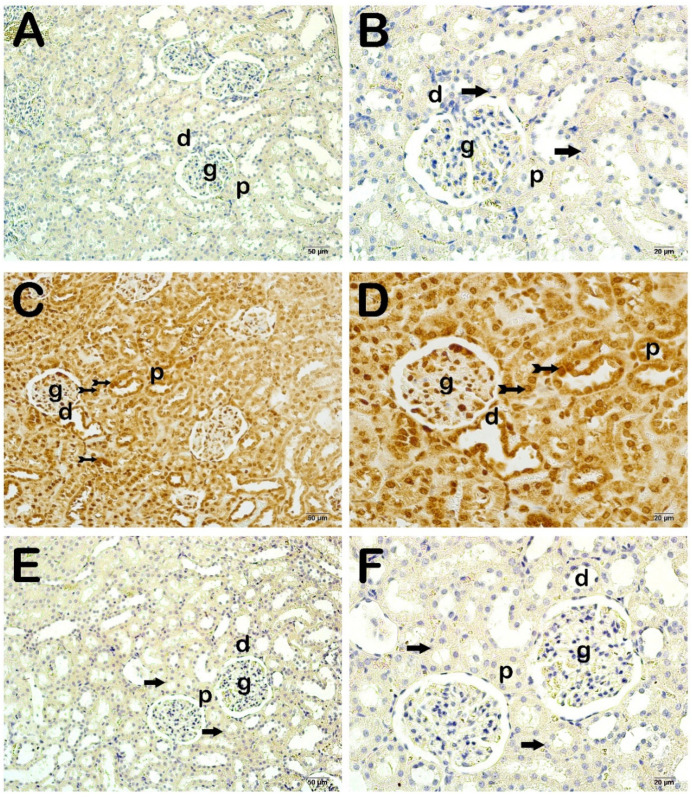
Representative light microscopic screenshot of sections of kidney tissue incubated with cleaved caspase-3 primary antibody. **A(x20)-B(x40):** In the sections of the control group, normal proximal and distal tubule epithelial cells are observed (arrow) (cleaved caspase-3 positivity score: 0 (0–1)). **C(x20)-D(x40):** Apoptotic proximal and distal tubule epithelial cells showing intense cleaved caspase-3 positivity are observed in the kidney tissue sections of the CLP application group (tailed arrow) (cleaved caspase-3 positivity score: 2 (2–3)). **E(x20)-F(x40**): In the kidney tissue sections of the FOS treatment group, apoptotic proximal and distal tubule epithelial cells showing cleaved caspase-3 positivity are observed to decrease (arrow) (cleaved caspase-3 positivity score: 0 (0–1)). Glomerulus (g), proximal tubule (p), distal tubule (d).

When the sections of kidney tissue incubated with cleaved caspase-3 were examined under a light microscope, there were normal proximal and distal tubule epithelial cells in the control group (Figure 2A,B; Table 5; *p* = 0.000; cleaved Caspase-3 positivity score: 0 (0–1)). However, we observed cleaved caspase-3 positive apoptotic proximal and distal tubule epithelial cells in the CLP-induced septic group (Figure 2C,D; Table 5; *p* = 0.000; cleaved caspase-3 positivity score: 2 (2–3)). In the FOS treatment group, we found that cleaved caspase-3 positivity was decreased in proximal and distal epithelial cells compared to the CLP-induced septic group (Figure 2E,F; Table 5; *p* = 0.000; cleaved caspase-3 positivity score: 0 (0–1)).

**Figure 3:**
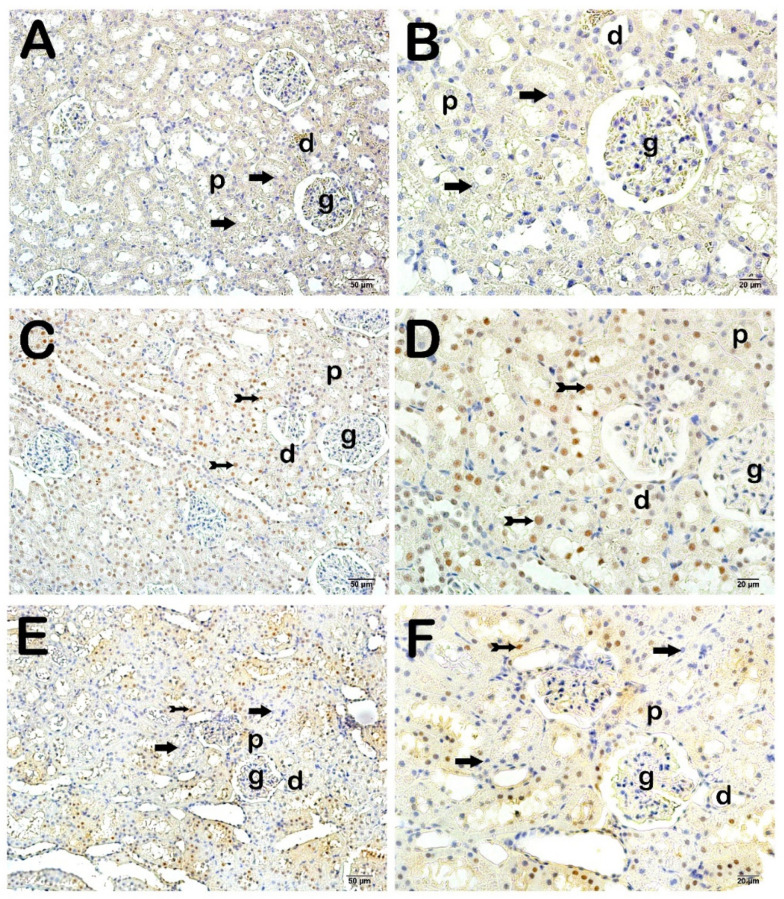
Representative light microscopic screenshot of kidney tissue sections incubated with 8-OHdG primary antibody. **A(x20)-B(x40):** Normally structured proximal and distal tubule epithelial cells are observed in the sections of the control group (arrow) (8-OHdG positivity score: 0 (0–0)). **C(x20)-D(x40):** In the kidney tissue sections of the CLP application group, proximal and distal tubule epithelial cells showing intense 8-OHdG positivity are observed (tailed arrow) (8-OHdG positivity score: 3(2–3)). **E(x20)-F(x40):** In the kidney tissue sections of the FOS treatment group, apoptotic proximal and distal tubule epithelial cells showing 8-OHdG positivity are observed to decrease (arrow) (8-OHdG positivity score: 0.5 (0–1)). Glomerulus (g), proximal tubule (p), distal tubule (d).

In kidney tissue sections incubated with the primary antibody of 8-hydroxy 2-deoxyguanosine (8-OHdG), the main product of DNA oxidation caused by free oxygen radicals in the nuclei, we found that 8-OHdG positivity was increased in the proximal and distal epithelial cells in the CLP-induced septic group compared to the control group (Figure 3A–D); Table 5; *p* = 0.000; 8-OHdG positivity score: 0 (0–0); 3 (2–3), respectively). On the other hand, we observed that 8-OHdG positive proximal and distal epithelial cells decreased in the Fos treatment group compared to the CLP-induced septic group (Figure 3C–F; Table 5; *p* = 0.000; 8-OHdG positivity score: 3 (2–3); 0.5 (0–1), respectively).

## 5. Discussion

Sepsis is defined as organ dysfunction due to dysregulation in immune response. Despite the potential involvement of many organs, the kidney represents a major target organ for inflammation and injury associated with sepsis [1,3,4,14]. In this study, where the antioxidant, anti-inflammatory, and anti-apoptotic effects of FOS were examined in kidney damage caused by CLP-induced sepsis, it was determined that FOS had regulatory effects on the damaged tissue and had no nephrotoxic effects.

Previous studies have shown that oxidative stress, pro-inflammatory and apoptosis mediators, and the tissue damage they cause can be improved with different treatments in the animal sepsis models [6,7,11,12,13]. However, research on FOS treatment is quite limited, and it is still unclear by what mechanism or mechanisms it exerts its antioxidant and anti-inflammatory effects. No study investigating its anti-apoptotic effect has been found. To our knowledge, our study is one of the first studies to evaluate these three parameters together.

It has been shown in the literature that many organ involvements and tissue damage, especially to the kidney, occur in sepsis due to oxidative stress and inflammation [1,5,6,7]. Similar to previous studies, the biochemical and histopathological results of this study showed that oxidative stress and inflammatory processes were triggered in the kidney due to CLP-induced sepsis. In the CLP + FOS group, it was determined that FOS plays a regulatory role in this damage, with its antioxidant and anti-inflammatory effects. Studies have reported that oxidative stress increases in sepsis and that one of the most important mechanisms playing a role in tissue damage is oxidative stress [6,7,14,15,24]. Malondialdehyde and glutathione are essential oxidative stress parameters. Studies by Allameh et al. and Topçu et al. have shown that MDA levels increase in sepsis [6,12]. Malondialdehyde is a biomarker of lipid peroxidation and is frequently used as an indicator of oxidative stress. Elevated MDA levels reflect the presence of oxidative damage to lipids and are indicative of an imbalance between the production of ROS and the body’s antioxidant defenses. Our study demonstrated a significant reduction in MDA levels within kidney tissue of CLP-induced septic animals following FOS treatment. This finding suggests that FOS may possess antioxidant properties capable of mitigating lipid peroxidation and the associated oxidative stress that occurs in sepsis. Oxidative stress-induced lipid peroxidation can damage cellular membranes and disrupt the integrity of lipid-rich structures within cells [25]. By reducing MDA levels, FOS may help preserve the structural integrity of renal cells and their membranes during sepsis. This preservation is crucial for maintaining cellular function and viability. The results of the study also supported our previous study [5].

Another oxidative stress parameter in this study is GSH, which provides protection against increased oxidative stress in the tissue. GSH is an important endogenous antioxidant that controls MDA levels that increase with oxidative stress and prevents tissue damage [6,12,23]. Previous studies have shown that while the MDA level increases in sepsis, the GSH level decreases [16,17,23]. Our study revealed that FOS treatment was associated with the preservation of GSH levels within the kidney tissue of CLP-induced septic animals. This finding suggests that FOS may have a protective effect on the antioxidant defense system in the kidneys during sepsis. The maintenance of GSH levels is crucial for combating oxidative stress. By preserving GSH levels, FOS may help enhance the ability of kidney tissue to neutralize harmful free radicals generated during sepsis. This, in turn, can contribute to the reduction of oxidative stress within the renal environment. GSH levels are intricately linked to both inflammation and apoptosis. Oxidative stress can activate pro-inflammatory pathways, while inflammation can deplete GSH levels. Additionally, GSH is involved in regulating apoptotic pathways. Fosfomycin’s ability to preserve GSH levels aligns with its anti-inflammatory and anti-apoptotic effects, proposing a multi-modal protective mechanism against kidney damage. Understanding the impact of FOS on GSH levels provides valuable insights into its potential therapeutic role in sepsis-associated kidney damage. Preserving GSH levels signifies an enhanced capacity to combat oxidative stress, which is a critical factor in maintaining cellular integrity and function during sepsis. As far as we have reviewed the literature, apart from these two studies, no study has been found showing the antioxidant activity of FOS [5]

Another important mechanism thought to play a role in sepsis is the triggering of the inflammatory pathway that develops due to damage caused by oxidative stress [7,9,12]. Pro-inflammatory cytokines, particularly interleukin-1 (IL-1) and interleukin-6 (IL-6), play a pivotal role in the pathogenesis of sepsis-induced organ damage, including kidney injury. Elevated levels of these cytokines are associated with an exaggerated and dysregulated immune response, which can contribute to tissue damage [7,9]. Various studies have investigated whether the inflammatory process, in addition to oxidative stress with different drugs, also contributes to nephrotoxicity in CLP-induced sepsis, and it has been shown that an increase in the expression of pro-inflammatory cytokines significantly increases tissue damage [7,9,19,23]. Our study demonstrated a significant reduction in the levels of IL-1 and IL-6 within kidney tissue of CLP-induced septic animals following FOS treatment. This finding suggests that FOS may have a regulatory effect on the pro-inflammatory cytokine response, attenuating the excessive release of IL-1 and IL-6 during sepsis. The reduction in IL-1 and IL-6 levels aligns with the potential anti-inflammatory properties of FOS. By dampening the pro-inflammatory cascade, FOS may help mitigate the damaging effects of an overactive immune response, such as the infiltration of immune cells into kidney tissue and the release of harmful inflammatory mediators. Excessive inflammation, as seen in sepsis, can lead to tissue injury and organ dysfunction [3]. The reduction in IL-1 and IL-6 levels within the kidney tissue shows that FOS may contribute to the preservation of renal function by minimizing inflammation-associated damage. The findings of the study are consistent with the results of a previous study showing the anti-inflammatory effects of FOS [5].

In our study, we observed noteworthy histological changes within the renal architecture of septic animals, and the effects of FOS on these alterations warrant discussion. Kidney damage in sepsis often manifests as tubular injury, characterized by tubular dilation, epithelial cell necrosis, and loss of tubular brush borders [23]. Our findings indicate that FOS treatment mitigated these histological changes in kidney tissue. Specifically, there was a reduction in tubular dilation, and the integrity of tubular epithelial cells appeared better preserved in the fosfomycin-treated group. This suggests that FOS may have a protective effect on the renal tubules, which are particularly susceptible to sepsis-induced damage. Interstitial inflammation, characterized by infiltrating immune cells in the renal interstitium, is a common feature of kidney injury in sepsis [26]. In our study, FOS treatment was associated with a decrease in interstitial inflammation in kidney tissue compared to untreated septic animals. This observation aligns with the potential anti-inflammatory properties of FOS. The reduction in interstitial inflammation is indicative of a possible attenuation of the exaggerated immune response that often exacerbates kidney damage in sepsis. The glomeruli, the functional units of the kidney, are critical for filtration and maintenance of renal function. Our histopathological assessment revealed that FOS treatment appeared to preserve the structural integrity of glomeruli in the septic kidneys. This finding suggests that FOS may play a role in protecting these crucial components of renal function. Taken together, the histopathological assessment of kidney tissue supports the notion that FOS has a beneficial impact on renal architecture in the context of CLP-induced sepsis. The observed reduction in tubular injury, interstitial inflammation, and preservation of glomerular structure collectively indicates a potential role for FOS in mitigating kidney damage and preserving renal function during sepsis. While further research, including mechanistic studies and clinical trials, is needed to confirm and elucidate these effects, our study provides valuable insights into the potential histological benefits of FOS as an adjunct therapy in the management of sepsis-associated kidney damage. The ability of FOS to ameliorate renal histopathological changes holds promise for improving outcomes in septic patients at risk of acute kidney injury.

In this study, we assessed the levels of 8-OHdG, a well-established biomarker of oxidative DNA damage, immunohistochemically in kidney tissue as a marker of DNA damage and oxidative stress. The presence of elevated 8-OHdG levels is indicative of oxidative damage to DNA, which is a critical factor in the pathogenesis of kidney injury in sepsis [27]. Our findings revealed that FOS treatment was associated with a significant reduction in 8-OHdG levels in the kidney tissue of CLP-induced septic animals. This observation suggests that FOS may have a protective effect against oxidative DNA damage in the kidneys during sepsis. The reduction in 8-OHdG levels is indicative of a potential role for FOS in mitigating DNA damage caused by ROS generated during sepsis.

In sepsis, apoptosis plays a significant role in organ injury, including kidney damage. Caspase-3 activation is a hallmark of apoptotic cell death, and assessing its activity provides insights into the extent of apoptosis within tissues [6,9,25]. Our study revealed a noteworthy finding that FOS treatment was associated with a reduction in caspase-3 activity within kidney tissue of CLP-induced septic animals. This reduction in caspase-3 activity suggests that FOS may possess anti-apoptotic properties, which could contribute to its protective effects against kidney damage in sepsis. By inhibiting caspase-3 activity, FOS may help preserve the structural and functional integrity of renal cells during sepsis.

Studies have shown that nephrotoxic damage may occur due to intensive treatment for sepsis [5,28,29]. Acute kidney injury caused by drugs used in sepsis is a serious pathology and complicates treatment efforts. In this regard, the selection of drugs to be used during sepsis management is highly important. It should be recommended to choose less nephrotoxic agents whenever possible. [10,13,28,29,30,31,32]. In the literature, it has been reported that FPS reduces the nephrotoxic effects of the adjacent drug when used in combination in sepsis; the number of studies showing that it is effective even when used alone in urosepsis is increasing [33,34,35,36,37]. Today, increasing antimicrobial resistance and underlying comorbid conditions of patients severely limit treatment options in sepsis [28,38,39,40]. It is clear that different options are needed.

However, there are also a number of limitations to this research. First, FOS was administered in a single dose, and its dose-dependent effect was not investigated. In addition, no clinical evaluation was performed because this study focused on oxidative stress, inflammation, and apoptosis mechanisms. Oxidant and antioxidant capacity markers superoxide dismutase, catalase, and glutathione reductase were not investigated. In addition, molecules such as tissue inhibitor metalloproteinase-2 and insulin-like growth factor binding protein-7, which indicate tubular damage, were not investigated. At the same time, other stages of the inflammatory process were not studied. In addition, our study should be supported by clinical studies addressing apoptotic processes and intracellular and mitochondrial Ca+2 levels. The optimal dosing regimens and timing of FOS administration in the context of sepsis management should also be addressed in future clinical studies.

## 6. Conclusions

This pilot study was conducted to determine the effectiveness of FOS, which we consider a promising new therapeutic agents in sepsis, in mitigating kidney damage caused by CLP, and to explain its mechanisms of action. In conclusion, this investigation has provided valuable insights into the potential therapeutic effects of fosfomycin on kidney damage induced by CLP-induced sepsis.

Summary: CLP-induced sepsis caused tissue damage primarily by increasing MDA levels in kidney tissue and also by depleting GSH, one of the elements of the antioxidant defense mechanism. Increased oxidative stress contributed to the exacerbation of this damage by activating pro-inflammatory systems. In addition, FOS prevented renal damage due to CLP-induced sepsis with its anti-apoptotic, antioxidant, and anti-inflammatory effects. With its anti-apoptotic effect, FOS exhibited nephrotoxic inhibition by reducing the cleaved caspase-3 positivity in apoptotic activity resulting from toxicity. With its anti-inflammatory effect, it especially reduced the levels of MDH, IL-1β, and IL6 and increased the total GSH level with its antioxidant effect.

## Figures and Tables

**Table 1:** Kidney Histopathological Score (KHDS).

Score	Findings
Renal Corpuscle Degeneration	
0	≤5%
1	Between 6–25%
2	Between 26–50%
3	≥50%
Tubular Necrosis	
0	≤5%
1	Between 6–25%
2	Between 26–50%
3	≥50%
Excessive Protein Exudation	
0	≤5%
1	Between 6–25%
2	Between 26–50%
3	≥50%
Intraluminar Necrotic Cellular Debris	
0	≤5%
1	Between 6–25%
2	Between 26–50%
3	≥50%

**Table 2:** Semi-quantitative analysis.

Score	
0	None (less than 5%)
1	Mild (between 6% and 25%)
2	Moderate (between 26% and 50%)
3	Severe (more than 51%)

**Table 3:** Biochemical analysis results.

	Groups									
	Control			GP1: CLP-Induced Septic			GP2: CLP + FOS			*p*
	Median	Minimum	Maximum	Median	Minimum	Maximum	Median	Minimum	Maximum	
**IL6** **(pg/gr tissue)**	69.1	53.8	92.3	79.0	55.1	103.4	63.6	60.6	99.6	0.707
**IL1β** **(pg/gr tissue)**	575.3	525.0	602.6	551.5	500.3	622.6	536.5	471.5	595.1	0.463
**GSH** **(mmol/gr tissue)**	6041	5452	6829	5953	4184	7178	7287 *	6281	7617	0.049
**MDA** **(nmol gr tissue)**	83.3	76.6	91.6	91.6	88.6	97.1	86.4	67.2	91.9	0.176

* It is statistically significantly higher in the GP2 group compared to the control group and the GP1 group.

**Table 4:** Kidney histopathological score results (KHDS, median (25–75%)).

Group	Renal Corpuscle Degeneration	Tubular Necrosis	Excessive Protein Exudation	Intraluminar Necrotic Cellular Debris	KHDS
**Control**	0 (0–0)	0 (0–0)	0 (0–0)	0 (0–0)	0 (0–0)
**CLP**	2 (2–2) ^a^	2 (2–3) ^a^	2 (1–2) ^a^	1 (1–2) ^a^	9 (9–10) ^a^
**CLP + FOS**	0 (0–1) ^b,c^	1 (1–2) ^a,c^	1 (1–1) ^a,c^	1 (1–1) ^a,d^	4.5 (4–6) ^a,c^

**^a^** *p* = 0.000; compared to control group, **^b^** *p* = 0.001; compared to control group, **^c^** *p* = 0.000; compared to CLP group, **^d^** *p* = 0.028; compared to CLP group, Kruskal–Wallis/Tamhane t2 test.

**Table 5:** Semi-quantitative analysis (KHDS, median (25–75%)).

Group	Cleaved Caspase-3 Positivity	8-OHdG Posivitiy
Control	0 (0–0)	0 (0–0)
CLP	2 (2–3) **^a^**	3 (2–3) **^a^**
CLP + FOS	0 (0–1) **^b,c^**	0.5 (0–1) **^b,d^**

**^a^** *p* = 0.000; compared to control group, **^b^** *p* = 0.005; compared to control group, **^c^** *p* = 0.000; compared to CLP group, **^d^** *p* = 0.029; compared to control group, Kruskal–Wallis/Tamhane t2 test.

## Data Availability

No datasets were generated or analyzed during the current study.
